# Henry J.M. Barnett (1922–2016)

**DOI:** 10.1007/s00415-024-12499-7

**Published:** 2024-08-09

**Authors:** J. van Gijn, L. J. Kappelle

**Affiliations:** University Medical Centre Utrecht, Utrecht, The Netherlands

**Fig. 1:**
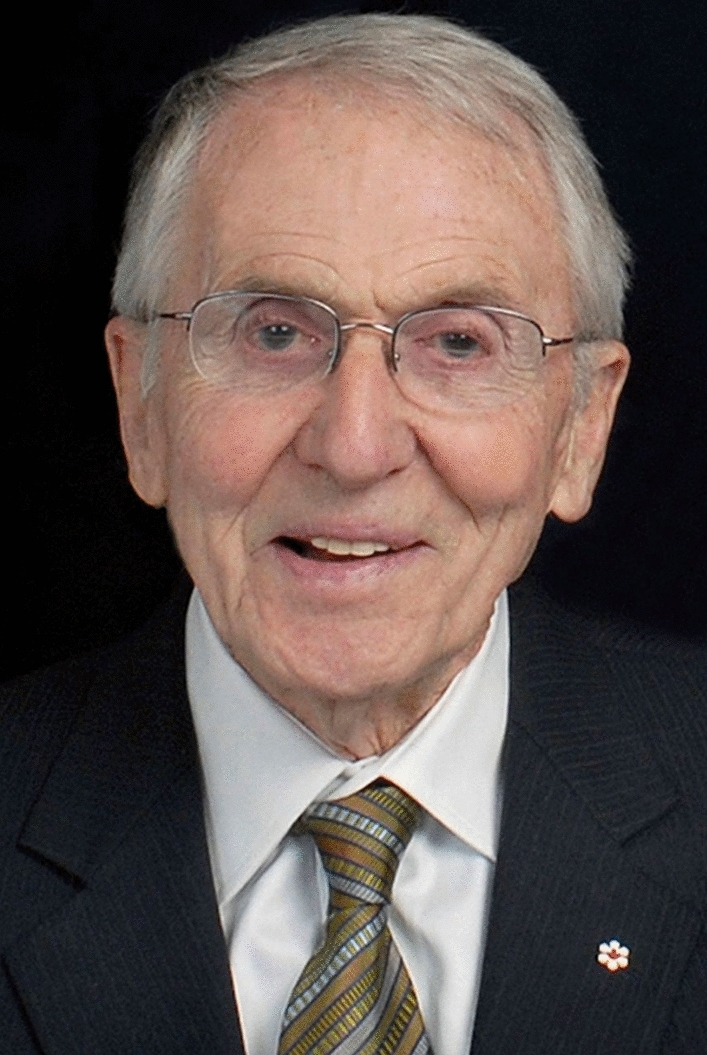
Henry J.M. Barnett

Henry J.M. Barnett, known to colleagues as ‘Barney’, was born in 1922, in Newcastle, United Kingdom (Fig. 1). His father, a clergyman, soon took his young family overseas to a parish in Toronto, Canada. Barney’s career in medicine was a compromise. Having skipped Sunday school as a 12-year-old, he met some ornithologists at the coastline of Lake Ontario; from that moment on, he became an avid birdwatcher, a passion he would retain all his life [1]. Initially, he wanted to study biology, but his father convinced him there was more of a future in medicine. In 1944, he graduated from the Toronto University School of Medicine. During internships at Toronto General Hospital (TGH), he had met nurse Kathleen (Kay) Gourlay. Not much later, she became his wife; they had four children [1]. He became a real ‘family man’ and a wonderful host for friends and colleagues.

Between 1946 and 1952, Barney more or less drifted into neurology. After 1 year of pathology and 2 years of internal medicine at TGH, he spent another 2 years alternating between neurology at TGH and the psychiatric unit at Wellesley Hospital. Astounding as well as somewhat annoying his seniors by his determination to practice only neurology in the future (“you will starve to death”), he spent two more years in the UK, at the National Hospital, Queen Square, London, and at the Radcliffe Infirmary in Oxford [1]. Back in Toronto, many years were filled by caring for patients, bedside teaching, and private consultations. He became a skilled neurologist, “contributing sparsely to the medical literature” [1]. Nonetheless, he became an expert on syringomyelia, especially the post-traumatic form; eventually, he co-authored a monograph on the subject [2].

In 1969, he moved from his alma mater to the University of Western Ontario (UWO) in London, Canada, prompted by the neurosurgeon Charles C. Drake (1920–1999), who had been there from his student days; they had become friends when both stayed in England. Together they convinced the UWO principals to establish a multidisciplinary Department of Clinical Neurosciences: chaired by a neurologist or a neurosurgeon (alternating every 5 years), integrated with neuroradiology and neuropathology [1]. In the course of time, young and ambitious researchers joined them, while Barney became especially interested in stroke. Why stroke, why in London, Ontario, and why at that time? Several factors can be identified: two predisposing and one decisive.

First, two other Canadian physicians had distinguished themselves in the field of cerebrovascular disease. Charles G. Drake, now Barney’s close colleague, was an expert in operative treatment of intracranial aneurysms, including types generally considered inoperable [3]. The other person was C. Miller Fisher, working in Montreal and later in Boston; in ischaemic stroke, he had cleverly identified the role of atherosclerotic lesions of the carotid artery, until then a ‘no man’s land’ between general pathology and neuropathology [4]. A second predisposing factor was the emergence of clinical epidemiology, a discipline allowing comparison of patient groups and evaluation of new or existing treatments. Following the trail of Richard Doll’s famous 1948 trial of streptomycin in tuberculosis, a department of clinical epidemiology had been established at the McMaster University in Hamilton, Ontario, halfway between London and Toronto. The decisive factor, however, for stroke research in general, was the invention, in the mid-1970s, of CT-scanning; this technique reliably distinguished between rupture and occlusion of brain arteries, a distinction that until then only autopsy had provided. Brain scanning opened the way to treatment, or at least to prevention of stroke. Barney and his team cast their nets widely, by assessing medical as well as surgical interventions in patients threatened by stroke, through three influential clinical trials.

Their first study, published in 1978, was pharmaceutical. Since “certain drugs … were known to have an inhibitory effect on platelet function and had had promising results among patients with cerebrovascular disease”, they tested the effect of a daily dose of 1300 mg of aspirin and 800 mg of sulfinpyrazone, together or alone, in 585 patients with transient ischaemic attacks or minor stroke. Sulfinpyrazone did not fulfil the expectations, but after more than 2 years, aspirin significantly reduced the risk of stroke or death, by 31% for all the patients. For women alone, the risk reduction was not significant [5]. True enough, later studies were to show that the alleged ‘aspirin resistance’ of women was a chance finding, that the dose of aspirin could be much lower, and that other antiplatelet agents existed, but the Canadian study opened the field.

The other studies involved two different surgical interventions. Both had already been introduced, but mainly on the basis of theory; they had not yet been subjected to the rigour of controlled clinical trials. Bypass surgery, from the territory of the external carotid artery, was becoming *en vogue* in patients with symptoms of cerebral ischaemia associated with ipsilateral occlusion or stenosis of the carotid or middle cerebral artery. Unfortunately, Barney’s multinational trial, published in 1985, failed to find a preventive effect on the occurrence of stroke or stroke-related death in 714 patients randomly assigned to surgical treatment, compared with 633 patients receiving best medical treatment [6]. Carotid endarterectomy in the neck had been an even more common operation with the purpose of preventing stroke, despite concerns about lack of evidence [7]. Barney, retired from hospital duties but continuing as head of a new research institute [8], launched in January 1988 a trial in 50 centres across Canada and the US; it was stopped after 3 years, after 659 patients with stenosis of 70–99% of the carotid artery had been randomised, because interim analysis showed a significant reduction for major or fatal ipsilateral stroke [9]. Almost simultaneously, this result was confirmed by a European trial of 2200 patients, which had been running from 1981 [10].

By the time of his death in 2016, Barney’s contributions to the management of stroke had made him world-famous and earned him honours such as inclusion in the Canadian Hall of Fame and honorary doctorates from the Canadian universities of Western Ontario and Dalhousie, as well from Utrecht (The Netherlands) and from Oxford (UK).
